# Adipose-derived mesenchymal stem cells from obese mice prevent body weight gain and hyperglycemia

**DOI:** 10.1186/s13287-021-02357-y

**Published:** 2021-05-06

**Authors:** Yicheng Qi, Wen Liu, Xiangsheng Wang, Nan Lu, Minglan Yang, Wei Liu, Jing Ma, Wei Liu, Wenjie Zhang, Shengxian Li

**Affiliations:** 1 Department of Endocrinology and Metabolism, Renji Hospital, School of Medicine, Shanghai Jiaotong University, No. 160 Pujian Road, Pudong New Area, Shanghai, 200127 China; 2 Department of Plastic and Reconstructive Surgery, Shanghai 9th People’s Hospital, School of Medicine, Shanghai Jiaotong University, Shanghai Key Laboratory of Tissue Engineering, No. 639 Zhizaoju Road, Huangpu Area, Shanghai, 200011 China

**Keywords:** Adipose-derived MSCs, Lipolysis, INSR, Obesity, Hyperglycemia

## Abstract

Changes that occur to the stem cell microenvironment with disease are a major consideration that may affect the behavior and potential therapeutic efficacy of mesenchymal stem cells (MSCs). The purpose of this study is to evaluate the effects of adipose-derived MSCs (ADSCs) from obese mice with hyperglycemia on body weight and glucose homeostasis. After 10 weeks of high-fat diet, mice were injected with phosphate-buffered saline (PBS) and ADSCs derived from normal mice (N-ADSCs) or obese mice (O-ADSCs), respectively. Mice fed with standard rodent chow were injected with PBS and served as normal controls. Obese mice treated with O-ADSCs showed less body weight gain than those receiving PBS or N**-**ADSCs. The mice that received ADSCs, especially O-ADSCs, also showed improvement in obesity-related hyperglycemia. In particular, the inguinal fat was reduced in obese mice receiving O-ADSCs compared with other groups, probably caused by the increased lipolysis of inguinal fat. Moreover, ADSC infusion restored insulin receptor (INSR) expression in the muscle of obese mice. Differential expression of the CD90 surface marker was slightly increased, while monocyte chemoattractant protein 1 (MCP-1) was reduced in O-ADSCs compared to N**-**ADSCs. These data provide a theoretical basis that autologous ADSCs from obese individuals may be more effective for treating obesity and related hyperglycemia.

**Supplementary Information:**

The online version contains supplementary material available at 10.1186/s13287-021-02357-y.

## Introduction

At present, there is a worldwide epidemic of obesity, which often leads to the development of type 2 diabetes mellitus (T2DM) and its associated medical and economic challenges. Over the last four decades, insulin resistance (IR), caused by obesity, has been identified as a major trigger of the T2DM epidemic in China [1]. To date, effective therapies for improving insulin resistance and curbing the development of obesity-related hyperglycemia have yet to be developed.

Mesenchymal stem cells (MSCs), which are characterized by their ability to self-renew and multipotentiality, have been identified in a number of tissues including bone marrow, fetal annexes, adipose, dental, and liver tissues [2]. The minimal characterization criteria for MSCs, as proposed by the International Society for Cellular Therapy in 2006, are as follows: (1) plastic-adherent; (2) expression of CD73, CD90, and CD105 and lack expression of CD45, CD34, CD14, CD11b, CD79α, CD19, and HLA-DR surface markers; and (3) multilineage differentiation potential toward adipocytes, osteocytes, and chondrocytes [3]. Of the many types of MSCs available, adipose-derived MSCs (ADSCs) are considered to have a number of advantages, including few ethical concerns (versus cells from fetal/neonatal tissues), ease of accessibility, and an abundance of cells from multiple sources [4]. Besides, previous evidences also confirmed the anti-obesity and anti-hyperglycemia benefits of ADSCs. Cao et al. revealed that ADSCs from healthy C57BL/6 mice reduced body weight and blood glucose levels in high-fat diet-induced obese mice. Further, the ADSC-injected mice displayed lower levels of macrophage (F4/80+) infiltration, interleukin-6 (IL-6), and nucleotide-binding oligomerization domain 2 (NOD2) in liver tissue, resulting in improved insulin resistance. Decreased macrophage infiltration was also discovered in pancreases after ADSC infusion, which may partially contribute to the protection of pancreatic β-cell mass [5]. In another report, it was found that the infusion of ADSCs alleviated hyperglycemia and insulin resistance in T2DM rats via restoration of glucose transporter-4 (GLUT4) and INSR expression on the cell membrane of the skeletal muscle, liver, and adipose tissue [6]. Others have shown that the infusion of ADSCs also has rapidly improved blood glucose levels (within 24 h) in T2DM rats which involved changes in the regulation of glycogen metabolism and gluconeogenesis via the AMPK signaling pathway [7]. Although all of these studies strongly suggest the utility of ADSCs in clinical applications, especially with regard to obesity and T2DM, the selection of allogeneic or autologous MSCs is still in question.

The allogeneic MSCs derived from donators are “off-the-shelf” cellular therapy. Although MSCs are previously identified as immune-privileged, it is also reported that allogeneic MSCs trigger local inflammation by allo-specific T-effector cells. Side effects are even accelerated after multiple infusions of allogeneic MSCs by boosting memory allo-response, which limits their further clinical application [8]. Fortunately, these problems were evitable in autologous MSC therapy. Autologous MSC therapy has higher acceptance and lower risk of infectious diseases. However, disease status always affects the function of MSCs. Obese adipose tissue is characterized by chronic inflammatory state, hypoxia, and metabolic disturbance [9]. These disorders may lead to impaired functions of ADSCs including multipotent differentiation ability, metabolism, and immunomodulation, which presumably reduce the curative effect of autologous ADSCs [10–12]. Thus, we wonder whether ADSCs isolated from normal control and obese mice showed different efficacy.

In the present study, we used a high-fat diet-induced mouse model of obesity and then compared the therapeutic efficacy of ADSCs derived from normal and obese donors on body weight and glucose homeostasis in obese mouse recipients. By taking this approach, we hope to better understand the underlying mechanisms affecting autologous ADSCs and their potential for use in T2DM therapy.

## Materials and methods

### Animals and experimental design

Since C57BL/6 mice are predisposed to high-fat diet-induced metabolic syndrome, we purchased 4-week-old male C57BL/6 mice from the Shanghai Jihui Laboratory of Animal Care for use in this study. After a 1-week period of adaptation to the environment, mice were fed a standard chow diet (10% of calories from fat) or a high-fat diet (HFD; 60% of calories from fat). Mice fed with the standard chow diet were used as normal controls. After 10 weeks, the HFD mice were randomized into 3 groups. Each group received a tail vein injection on days 0, 4, and 9. Group 1 received phosphate-buffered saline (PBS) (*n*=5); group 2 received 5×10^5^ ADSCs from normal control mice (N-ADSCs) (*n*=5), and group 3 received 5×10^5^ ADSCs from obese mice (O-ADSCs) (*n*=6). Body weight and blood glucose levels were continuously monitored and percent change calculated. Two months after tail vein infusion, intraperitoneal glucose tolerance (IPGTT) and insulin releasing test (IRT) were performed after a 12-h fast. At the end of the experiment, mice were anesthetized with 10% chloral hydrate, and dissected tissues were frozen in liquid nitrogen immediately after sacrifice. Inguinal, epididymal fat pads were weighed after excision.

### ADSC isolation and culture

Fresh inguinal fat was isolated from normal control and obese mice and washed with Dulbecco’s phosphate-buffered saline (D-PBS, Gibco). Adipose tissues were cut into small pieces and then digested with 2mg/mL type I collagenase (Sigma-Aldrich) for 30 min at 37°C on a shaker. Cells were resuspended after centrifugation at 1500 rpm for 5 min and then filtered through a 40-μm nylon filter mesh (BD Falcon) to remove any tissue residue. After washing twice, cells were resuspended in alpha-MEM medium (Gibco) containing 10% fetal bovine serum (Gibco) and seeded in T75 tissue culture flasks (BD Falcon). Cells were cultured in a humidified 5% CO_2_ incubator at 37°C. Floating cells were removed after 24 h and the media changed after 3 days. At 90% confluence, cells were detached with 0.25% trypsin-EDTA and passaged.

### Intraperitoneal glucose tolerance and insulin releasing test

For IPGTT, all mice received an intraperitoneal injection of glucose (2g/kg) after an overnight fast. At 0, 15, 30, 60, 90, and 120 min after glucose load, blood glucose levels were measured with a glucometer (Roche). At 0, 15, and 30 min, 0.1-mL blood samples were collected from the orbital venous plexus. Immediately after centrifugation, plasma was obtained and promptly stored at −80°C. Insulin assay was subsequently performed by ELISA (Alpco) according to the manufacturer’s protocol. HOMA-IR index was calculated by the equation: HOMA-IR index = (FBG [in mmol/L] × FINS [in units/L])/22.5.

### RNA extraction and quantitative RT-PCR

Total RNA was extracted from liver, muscle, and adipose tissues using the RNeasy Kit (Qiagen), followed by converting into the first-strand cDNA with the cDNA synthesis kit (Takara). Quantitative RT-PCR was performed using SYBR Master Mix (Takara) and a LightCycler 480 System (Roche). The quantity of mRNA was normalized to β-actin in the liver and muscle and to 36B4 in adipose, respectively. The primer sequences used for this study are provided in Supplement Table 1.

### Western blot analysis

Total protein was extracted from liver, muscle, and adipose tissues for Western blot analysis using a standard protocol. Thirty micrograms of protein was separated by SDS-PAGE and then transferred to a PVDF membrane (Millipore Corp). After blocking, the membranes were incubated overnight at 4°C with primary antibodies to Scd-1 (1:2000), Adr-β3 (1:2000), INSR (1:2000), and GAPDH (1:2000) (Abcam). Next, these membranes were incubated with horseradish peroxidase-conjugated secondary antibody (Cell Signaling Technology). Protein bands were illuminated using ECL Prime Western Blotting Detection Reagent (GE Healthcare). This assay was repeated at least three times and a representative Western blot was chosen for the figures.

### Flow cytometry

Cell suspensions containing 1×10^6^ cells were incubated in 100μL of diluted FITC-conjugated rat anti-mouse CD44, CD90, CD105, and CD45 antibodies (BD Biosciences) for 30 min at 4°C. After surface staining, cells were washed and analyzed by flow cytometry (BD Biosciences). The appropriate isotype antibodies were used as controls.

### Statistical analyses

Statistical analysis was performed using SPSS version 25 and Graph Pad Prism 8. Data were expressed as mean±SD. Student’s *t* test or ANOVA was performed to analyze the differences among groups. The area under the curve (AUC) of the IPGTT and IRT were calculated. *p*<0.05 was considered statistically significant.

## Results

### Generation of obese mice

**Fig. 1:**
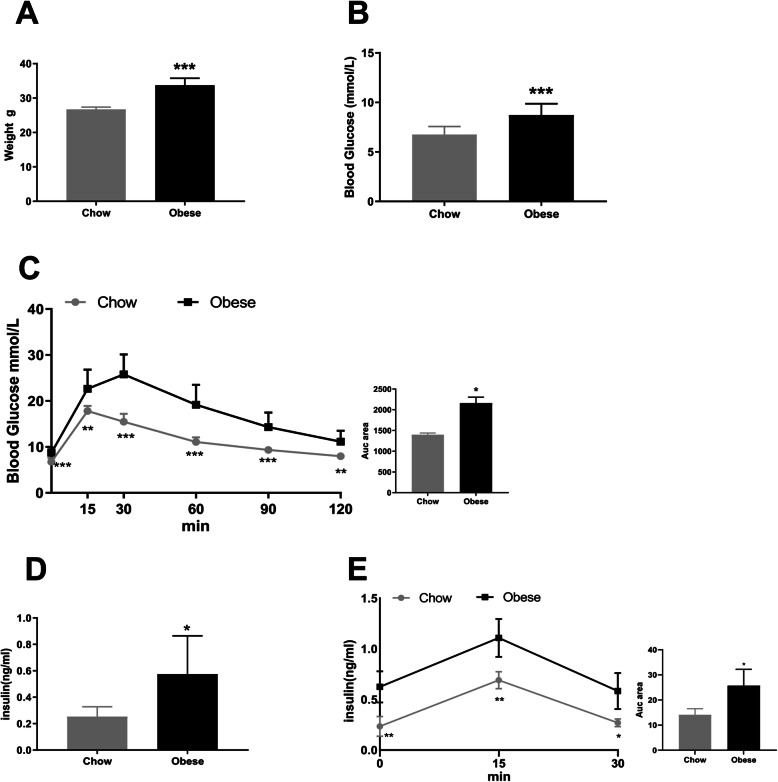
Generation of obese mice. **a** Body weights. **b** Fasting blood glucose. **c** IPGTT. **d** Insulin levels. **e** IRT in C57BL/6 mice fed with a standard chow diet or a high-fat diet. Data are mean±SD. *p<0.05, **p<0.01, ***p<0.001

The obese mouse model was established by feeding the mice with HFD. Compared to the normal control group fed standard chow, body weight in the animals receiving the HFD increased significantly after 10 weeks (Fig. 1a, 26.72±0.67g vs. 33.73±2.02g, *p*<0.001). In addition, fasting blood glucose (FBG) levels in the obese mice were significantly higher than in the normal controls (Fig. 1b, 8.72±1.15mmol/L vs. 6.75±0.82mmol/L, *p*<0.001). Further, obese mice had impaired glucose tolerance in the IPGTT. HFD also induced insulin resistance with higher insulin levels (Fig. 1c). These results indicate that our obese mouse model is appropriate for use in our study.

### ADSC infusion reduced body weight

**Fig. 2:**
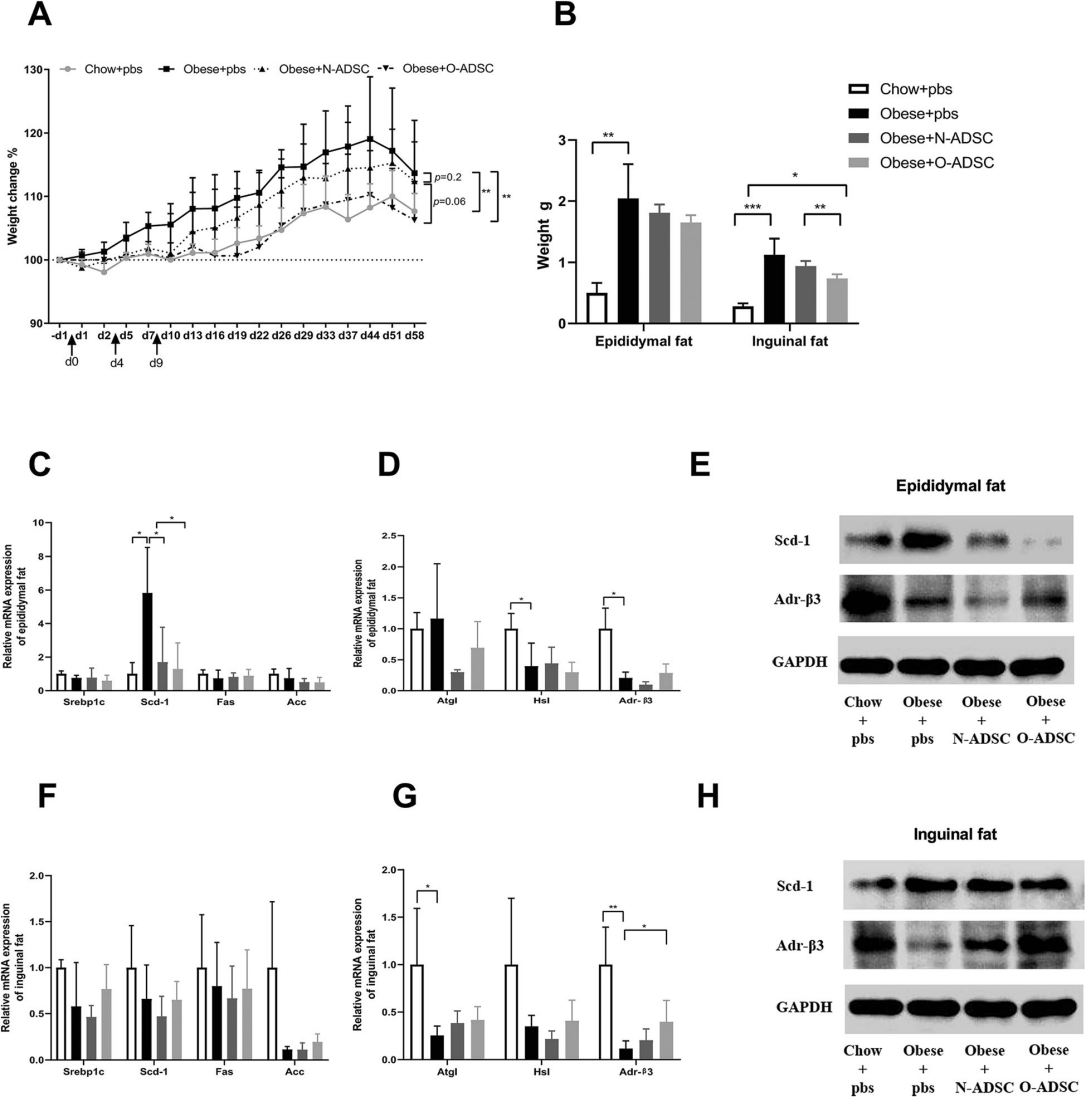
The effect of ADSC infusion on body weights and fat mass. **a** Percentages of body weight changes after ADSC injection. **b** Epididymal fat weights and inguinal fat weights after ADSC injection. **c**, **d** Effect of ADSC infusion on mRNA levels of adipogenesis genes and lipolysis genes in epididymal fat. **e** Scd-1 and Adr-β3 protein levels in epididymal fat after ADSC or PBS infusion. **f**, **g** Effect of ADSC infusion on mRNA levels of adipogenesis genes and lipolysis genes in inguinal fat. **h** Scd-1 and Adr-β3 protein levels in inguinal fat after ADSC or PBS infusion. Quantitative RT-PCR and Western blot experiments were repeated at least three times. Data are mean±SD. *p<0.05, **p<0.01, ***p<0.001

To explore the effect of ADSCs on body weight, we injected ADSCs into obese mice via the tail vein. Interestingly, we found that injection of O-ADSCs significantly slowed the increase in weight of mice compared to those injected with PBS or N-ADSCs (Fig. 2a). We next measured the weights of epididymal fat and inguinal fat at the end of the observation period and found that infusion of ADSCs failed to influence the weight of epididymal fat pads. However, inguinal fat pad weight was significantly reduced in mice injected with O-ADSCs as compared to mice injected with N-ADSCs or PBS (Fig. 2b). We next evaluated the expression of genes associated with adipogenesis and lipolysis in adipose tissues to determine the probable cause of the observed reduction in weight. As shown in Fig. 2c–e, obese mice injected with both N- and O-ADSCs were observed to downregulate stearoyl-CoA desaturase 1 (Scd-1) at both the transcript and protein levels in epididymal fat, although mice injected with O-ADSCs showed the most dramatic decline. Meanwhile, O-ADSCs restored the β3-adrenergic receptor (Adr-β3) expression in inguinal fat of obese mice (Fig. 2f–h). These data suggest that ADSCs, especially O-ADSCs, have a protective effect in obesity by partially increasing lipolysis in inguinal adipose tissue.

### Infusion of ADSCs improved glucose homeostasis and insulin sensitivity

**Fig. 3:**
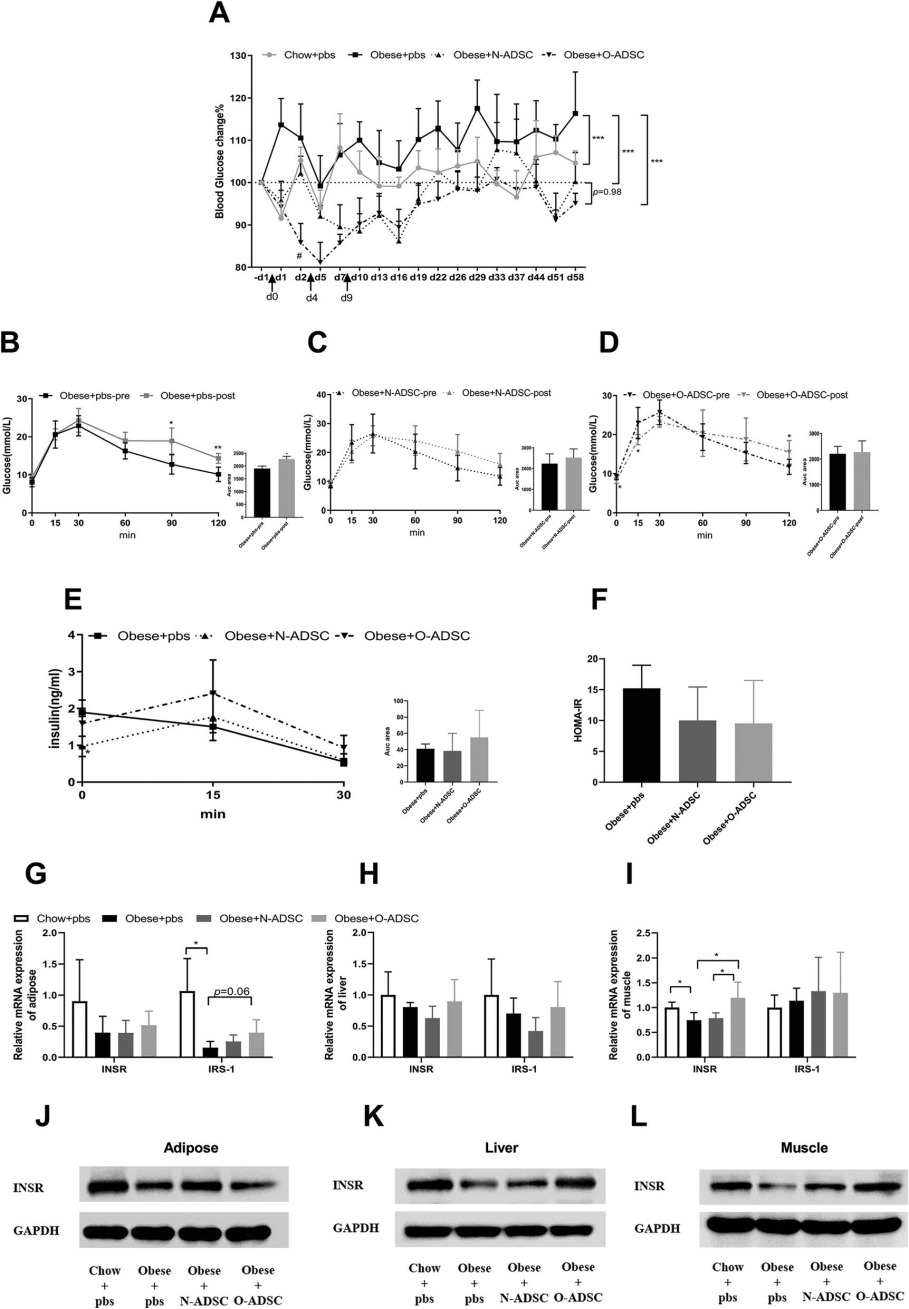
The effect of ADSC infusion on blood glucose levels and insulin sensitivity. **a** Percentages of blood glucose changes after ADSC injection. **b**–**d** IPGTT and area under the curve before and after PBS, N-ADSC, or O-ADSC injection. **e** Insulin releasing test before and after PBS, N-ADSC, or O-ADSC injection. **f** HOMA-IR after ADSC injection. **g**–**i** Effect of ADSC infusion on the mRNA expression of INSR and IRS-1 in insulin target tissues. **j**–**l** Effect of ADSC infusion on the protein levels of INSR in insulin target tissues. Quantitative RT-PCR and Western blot experiments were repeated at least three times. Data are mean±SD. *p<0.05, **p<0.01, ***p<0.001

It has been indicated that obesity has a high risk for insulin sensitivity, we next examined whether infusion of ADSCs has an impact on glucose homeostasis. Obese mice receiving N-ADSCs or O-ADSCs had lower blood glucose levels than the controls, but the effect of treatment with O-ADSCs was more dramatic by day 2 after the first injection (Fig. 3a).

After 2 months of successive HFD feeding, the glucose tolerance became even more impaired in the obese mice (Fig. 3b). However, the infusion of ADSCs attenuated the decline in glucose tolerance, especially at the 15-min time point after glucose loading in the O-ADSCs group (Fig. 3c, d). Further, insulin response after glucose load was declined in the PBS group, but remained normal in both ADSC-treated groups (Fig. 3e). Obese mice receiving ADSCs also tended to display improved insulin sensitivity, but showed no statistically significant difference between the PBS and ADSCs groups (Fig. 3f).

To understand the underlying mechanisms, we measured the gene expression of insulin downstream molecules in adipose, liver, and muscle tissues (Fig. 3g–l). We found that the mRNA expression and protein levels of INSR in the muscle were dramatically elevated in obese mice receiving infusions of N-ADSCs and O-ADSCs, compared to those treated with PBS, but mice receiving O-ADSCs showed higher levels. In addition, INSR protein levels were increased in the liver after treatment with ADSCs. Taken together, these results demonstrated that ADSCs, especially O-ADSCs, improve glucose homeostasis and insulin sensitivity in obese mice.

## Discussion

This study revealed, for the first time, that O-ADSCs are a more effective cell-based therapy for treating obesity and deranged glucose homeostasis in HFD mice than N-ADSCs. Further, an improvement in body weight was accompanied by an increase in lipolytic gene expression in inguinal fat tissue after treatment with O-ADSCs. Moreover, O-ADSC treatment also tended to attenuate insulin resistance which coincided with the restoration of INSR expression in the muscle.

In this study, ADSCs were isolated from inguinal fat, a type of subcutaneous adipose tissue (SAT), which is less influenced by obesity and T2DM than visceral adipose tissue (VAT) [13]. In previous studies, the effect of ADSC treatment on body weight has been shown to be associated with disease progression. ADSCs reduce body weight in obese mice fed a HFD for 20 weeks with a negligible effect on T2DM, while body weight gradually increases during the later phase of diabetes [5, 6, 14]. In our study, we infused N**-**ADSCs into obese mice, as an early intervention, after 10 weeks of HFD feeding with no effect on body weight. In contrast, the infusion of O-ADSCs retarded body weight gain compared to the PBS and N**-**ADSCs groups, although a number of previous studies suggest that detrimental changes to the microenvironment in obese tissue impair the function of ADSCs [10]. It has been shown that MSCs are often quiescent and that their immunosuppressive properties are induced by inflammatory cytokines such as interferon-γ (IFNγ), interleukin-17 (IL-17), and tumor necrosis factor (TNF) in the microenvironment [15]. In addition, hypoxia may also enhance the function of MSCs via increased expression of IL-6, vascular endothelial growth factor (VEGF), and chemokines [16]. The data we report here and that of previous studies suggest that changes in the microenvironment might activate MSC function. Thus, it is important to consider factors, such as the location where ADSCs are obtained, the time between isolation and infusion, and cell activity/function, before MSC therapy.

Obesity is characterized by excessive fat accumulation [17]. Consistent with the previous report [5], we found no differences in the weights of the liver (data not show) and epididymal fat among control, N-ADSCs, and O-ADSCs groups in our study. Interestingly, we found that infusion of O-ADSCs decreased inguinal fat pad weight compared to treatment with PBS or N-ADSCs. Another study revealed that the anti-obesity effect of ADSCs was due to decreased lipogenesis and increased lipolysis through hormone-sensitive lipase activation and acetyl-CoA carboxylase1 suppression [18]. In the present study, we found that both N-ADSC and O-ADSC infusion inhibited lipogenesis by reducing Scd-1 expression of epididymal adipose tissue, while only O-ADSC treatment activated lipolysis by enhancing Adr-β3 expression in inguinal adipose tissue.

Weight loss in obese and overweight patients with T2DM is associated with improvement in hyperglycemia [19]. As with previous research, our data suggest that ADSCs also significantly improve blood glucose levels in obese mice, especially in the O-ADSCs group. Animal studies have demonstrated that infusion of ADSCs reduces blood glucose levels via multiple mechanisms, including promotion of insulin production, improvement of insulin resistance, and regulation of hepatic glucose metabolism [20]. We found that ADSCs prevented the decline in insulin response to glucose load in HFD mice, which was related to the restoration of INSR expression in the muscle, especially seen in HFD mice treated with O-ADSC infusion. The muscle is considered as a major target organ of insulin action largely involved in systemic insulin resistance. Considering that glucose uptake of the muscle accounts for approximately 80% of insulin-mediated glucose utilization, the muscle plays a key role in whole-body glucose homeostasis [21]. Therefore, the restoration of INSR in the muscle may partially explain the relative advantage of O-ADSCs over N-ADSCs in glycemic control.

We further characterized the properties of the two types of ADSCs at the cytological level. As supplement Fig. 1 shown, both N-ADSCs and O-ADSCs were fibroblast-like and normal growth. Both ADSCs displayed similar phenotypes with CD44, CD105, and CD45 expression, except that O-ADSCs expressed a slightly higher level of CD90 as compared to that of the N-ADSCs. In addition, there was a downregulation of monocyte chemoattractant protein 1 (MCP-1) and interleukin-1β (IL-1β) in O-ADSCs as compared with N-ADSCs, which may partially explain the different effects of the two cell types. However, these data are not sufficient to identify the whole underlying mechanism. Further studies need to be conducted to clarify the cause of the phenomenon we have observed.

In conclusion, our results, for the first time, have provided evidence that O-ADSCs better reduced body weight and blood glucose levels than N-ADSCs. Further, the mechanism may be that O-ADSCs regulated the lipid metabolism of adipose tissue and improved insulin resistance of the skeletal muscle. The analysis of surface markers showed that O-ADSCs expressed slightly higher levels of CD90 while lower levels of MCP-1 than N-ADSCs. These findings demonstrate that O-ADSC infusion may be ideally used for controlling obesity and its related hyperglycemia and further support the development of autologous ADSC-based therapy for an obesity-related T2DM.

## Supplementary Information

**Additional file 1: Supplement Table 1.** Primer sequences used in quantitative RT-PCR.

**Additional file 2: Supplement Figure 1.** Both N-ADSCs and O-ADSCs were fibroblast-like and normal growth.

## Data Availability

The datasets used and/or analyzed during the current study are available from the corresponding authors on reasonable request.

## Abbreviations

MSCs: Mesenchymal stem cells
ADSCs: Adipose-derived MSCs
PBS: Phosphate-buffered saline
N-ADSCs: ADSCs from normal control mice
O-ADSCs: ADSCs from obese mice
T2DM: Type 2 diabetes mellitus
IR: Insulin resistance
INSR: Insulin receptor
IRS-1: Insulin receptor substrate 1
MCP-1: Monocyte chemoattractant protein 1
IL-6: Interleukin-6
NOD2: Nucleotide-binding oligomerization domain 2
GLUT4: Glucose transporter-4
HFD: High-fat diet
IPGTT: Intraperitoneal glucose tolerance
IRT: Insulin releasing test
D-PBS: Dulbecco’s phosphate-buffered saline
AUC: Area under the curve
FBG: Fasting blood glucose
Scd-1: Adipogenesis gene stearoyl-CoA desaturase 1
Adr-β3: Beta3-adrenergic receptor
SAT: Subcutaneous adipose tissue
VAT: Visceral adipose tissue
IFNγ: Interferon-γ
IL-17: Interleukin-17
TNF: Tumor necrosis factor
VEGF: Vascular endothelial growth factor
IL-1β: Interleukin-1β
