# Supplementary material for: Adipose-derived mesenchymal stem cells from obese mice prevent body weight gain and hyperglycemia
Source: Stem Cell Res Ther. 2021 May 6;12:277. doi: 10.1186/s13287-021-02357-y (PMC8101155; doi:10.1186/s13287-021-02357-y)
Supplement: Supplementary file 1 — Additional file 1: Supplement Table 1. Primer sequences used in quantitative RT-PCR. [file 13287_2021_2357_MOESM1_ESM.docx]

Supplement Table. 1. Primer sequences used in quantitative RT-PCR

| Genes | Forward primer (5’- 3’) | Reverse primer (5’- 3’) |
| --- | --- | --- |
| β-actin | GTGACGTTGACATCCGTAAAGA | GCCGGACTCATCGTACTCC |
| 36B4 | GAAACTGCTGCCTCACATCCG | GCTGGCACAGTGACCTCACACG |
| Srebp1c | GATGTGCGAACTGGACACAG | CATAGGGGGCGTCAA ACAG |
| Scd-1 | ACTGTGGAGACGTGTTCTGGA | ACGGGTGTCTGGTAGACCTC |
| Fas | TATCAAGGAGGCCCATTTTGC | TGTTTCCACTTCTAAACCATGCT |
| Acc | CTGACGTATACTGAACTGGTG TTGGATG | TTTCCAGGCTACCATGCCAATCTC |
| Atgl | GCATCCAGTTCAACCTTCGC | GGGTTGGTTCAGTAGGCCAT |
| Hsl | TTCTCCAAAGCACCTAGCCAA | TGTGGAAAACTAAGGGCTTGT TG |
| Adr-β3 | AGA AAC GGC TCT CTG GCT TTG | TGG TTA TGG TCT GTA GTC TCG G |
| INSR | TCAAGACCAGACCCGAAGATT | TCTCGAAGATAACCAGGGCATAG |
| IRS-1 | TCTACACCCGAGACGAACACT | TGGGCCTTTGCCCGATTATG |
| MCP-1 | CAGCCAGATGCAGTTAACGC | GCCTACTCATTGGGATCATCTTG |
| IL-1β | GCAACTGTTCCTGAACTCAACT | ATCTTTTGGGGTCCGTCAACT |
| IL-6 | TAGTCCTTCCTACCCCAATTTCC | TTGGTCCTTAGCCACTCCTTC |
| TNF-α | TTGGTCCTTAGCCACTCCTTC | GCTACGACGTGGGCTACAG |
